# High-Detectivity Organic Photodetector with InP Quantum Dots in PTB7-Th:PC_71_BM Ternary Bulk Heterojunction

**DOI:** 10.3390/polym17162214

**Published:** 2025-08-13

**Authors:** Eunki Baek, Sung-Yoon Joe, Hyunbum Kang, Chanho Jeong, Hyunjong Lee, Insung Choi, Sohee Kim, Sangjun Park, Dongwook Kim, Jaehoon Park, Jae-Hyeon Ko, Gae Hwang Lee, Youngjun Yun

**Affiliations:** 1 School of Semiconductor∙Display Technology, Hallym University, Chuncheon 24252, Republic of Korea; 2 Samsung Advanced Institute of Technology (SAIT), Samsung Electronics, Suwon 16678, Republic of Korea

**Keywords:** organic photodiode (OPD), bulk heterojunction (BHJ), InP quantum dots, photoplethysmography, specific detectivity, low dark current, polymers

## Abstract

Organic photodetectors (OPDs) offer considerable promise for low-power, solution-processable biosensing and imaging applications; however, their performance remains limited by spectral mismatch and interfacial trap states. In this study, a highly sensitive polymer photodiode was developed via trace incorporation (0.8 wt%) of InP/ZnSe/ZnS quantum dots (QDs) into a PTB7-Th:PC_71_BM bulk heterojunction (BHJ) matrix. This QD doping approach enhanced the external quantum efficiency (EQE) across the 540–660 nm range and suppressed the dark current density at −2 V by passivating interface trap states. Despite a slight decrease in optical absorption at the optimized composition, the internal quantum efficiency (IQE) increased significantly from ~80% to nearly 95% resulting in a net EQE improvement. This suggests that QD incorporation improved charge transport without compromising charge separation efficiency. As a result, the device achieved a specific detectivity (D*) of 1.8 × 10^13^ Jones, representing a 93% improvement over binary BHJs, along with an ultra-low dark current density of 7.76 × 10^−10^ A/cm^2^. Excessive QD loading, however, led to optical losses and increased dark current, underscoring the need for precise compositional control. Furthermore, the enhanced detectivity led to a 4 dB improvement in the signal-to-noise ratio (SNR) of photoplethysmography (PPG) signals in the target wavelength range, enabling more reliable biophotonic sensing without increased power consumption. This work demonstrates that QD-based spectral and interfacial engineering offers an effective and scalable route for advancing the performance of OPDs, with broad applicability to low-power biosensors and high-resolution polymer–QD imaging systems.

## 1. Introduction

Solution-processed bulk heterojunction (BHJ) organic photodiodes (OPDs) are attracting increasing attention as key components of next-generation wearable and biomedical sensors, primarily because of their low-temperature processability and microwatt-level power consumption. The commonly used PTB7-Th:PC_71_BM blend with a 1:1.5 wt ratio exhibits excellent hole mobility and broadband optical absorption. However, its practical performance is limited by several factors, including insufficient absorption in the 530–610 nm green–orange region, which is essential for accurate photoplethysmography (PPG) signal detection. In addition, defects at the polymer–fullerene interface lead to increased dark current density (J_d_), whereas the resulting noise further degrades the specific D* and the signal-to-noise ratio (SNR) of PPG measurements. Several studies have aimed to address this challenge [1,2,3,4,5].

One approach to improving the performance of BHJ OPDs involves controlling the film microstructure through careful selection of the deposition technique. The nanoscale morphology of polymer:fullerene blends strongly influence key optoelectronic properties, including absorption characteristics and photoluminescence (PL) quenching efficiency. For instance, Todor-Boer et al. reported that PBTTT:PCBM films fabricated via convective self-assembly (CSA) exhibit significantly different domain structures compared to spin-coated films, resulting in marked variations in optical absorption and PL quenching due to differences in phase separation and molecular ordering [6]. These findings underscore the importance of processing-induced morphological control in optimizing charge generation and transport in OPD systems. Another promising strategy is the incorporation of a third light-absorbing component, such as QDs, into the polymer:fullerene BHJ matrix. This compositional engineering approach can enhance spectral absorption in underrepresented regions and mitigate interfacial trap states without disrupting the BHJ morphology. In particular, cadmium-free InP/ZnSe/ZnS QDs exhibit a strong excitonic absorption near 610 nm and benefit from robust surface passivation due to their multi-shell structure. Previous studies have investigated the use of QDs in combination with photodiodes to improve performance [5,7,8,9,10,11,12,13,14], and trace amounts of InP/ZnSe/ZnS QDs have been shown to enhance the photocurrent in PTB7-Th systems [8,15]; however, the quantitative effect of QD content on PPG sensing performance remains unclear. Previous studies have also reported on planar-stacked mercury telluride (HgTe) QD/poly[4,8-bis(5-(2-ethylhexyl) thiophen-2-yl)benzo[1,2-b:4,5-b′]dithiophene-alt-3-fluorothieno[3,4-b]thiophene](PBDB- T):Y6 photodetectors [16]. While II–VI QDs, such as HgTe QDs and lead sulfide (PbS) QDs, perform well in the short-wave infrared (SWIR) region above 900 nm, they present issues of toxicity due to mercury and lead and have limitations in terms of sensitivity to visible light.

In this study, we systematically prepared six ternary blends with a fixed PTB7-Th to PC_71_BM weight ratio and varying InP/ZnSe/ZnS QDs content. PL spectroscopy revealed that both the QD and PTB7-Th emissions were fully quenched, indicating efficient exciton transfer to the BHJ interface and suppression of radiative recombination. Given that increasing the QD concentration results in a trade-off between absorption enhancement and optical light loss, attributed to reabsorption and scattering, we determined the optimal QD content to be 0.8 wt%. This optimized content effectively balances the PL quenching, spectral enhancement, and trap suppression. These findings complement the existing interfacial engineering approaches in near-infrared-based devices and provide a solution-process-compatible path toward high-sensitivity, low-noise OPD.

## 2. Materials and Methods

### 2.1. Materials

**Figure 1:**
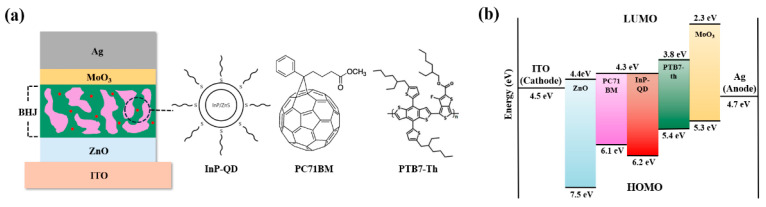
(**a**) Device structure of our OPD and chemical structures of BHJ. (**b**) Energy level alignment of BHJ.

This study employed InP/ZnSe/ZnS core–shell–shell QDs as photoactive additives [17,18]. The donor polymer used was Poly[4,8-bis(5-(2-ethylhexyl)thiophen-2-yl)benzo [1,2-b;4,5-b’]dithiophene-2,6-diyl-alt-(4-(2-ethylhexyl)-3-fluorothieno[3,4-b]thiophene-)-2-carboxylate-2-6-(PTB7-Th), comprising alternating donor (BDT) units and electron-deficient fluorinated thienothiophene units. This molecular architecture facilitates strong intramolecular charge transfer, resulting in broadband absorption from 300 to 750 nm and a hole mobility on the order of 10^−4^ cm^2^ V^−1^ s^−1^. In addition, the energy levels of PTB7-Th (HOMO ≈ −5.4 eV, LUMO ≈ −3.8 eV) are well aligned with those of fullerene acceptors. As the electron acceptor, we used 3′H-Cyclopropa[7,25][5,6]fullerene-C70-D5h(6)-3′-butanoic acid, 3′-phenyl-, methyl ester(PC_71_BM), a C_70_-derived fullerene functionalized with a solubilizing methyl-ester side chain. PC_71_BM features a deep LUMO level (≈−4.3 eV) and an electron mobility on the order of a few ×10^−3^ cm^2^ V^−1^ s^−1^ [19,20,21], which promotes efficient exciton dissociation and electron transport. Figure 1 presents a schematic of the inverted all-polymer photodiode incorporating InP/ZnSe/ZnS QDs, along with the chemical structures of each active layer material. The device is built on an indium tin oxide (ITO) substrate and comprises, from bottom to top, a ZnO electron transport layer, a BHJ photoactive layer composed of PTB7-Th:PC_71_BM:InP/ZnSe/ZnS QDs, a molybdenum trioxide (MoO_3_) hole-transport layer, and a silver top electrode. The molecular structures of the PTB7-Th, PC_71_BM, and the InP/ZnSe/ZnS QDs are shown along with the device stack.

### 2.2. Methods

**Figure 2:**
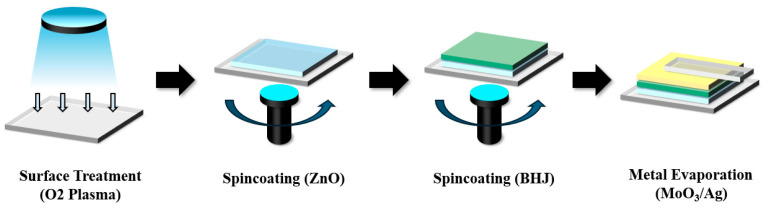
Schematic of device fabrication.

Figure 2 shows the experimental process schematically. To prepare the ZnO electron transport layer, 1 g of zinc acetate dihydrate (Sigma-Aldrich, St. Louis, MO, USA) was dissolved in 10 mL of 2-methoxyethanol (Sigma-Aldrich, St. Louis, MO, USA) and stirred at room temperature. Subsequently, 0.27 mL of ethanolamine (Sigma-Aldrich, St. Louis, MO, USA) was added, and the solution was stirred overnight to yield a clear precursor solution. This solution was filtered through a 0.2 µm PTFE filter and used immediately. The ITO-coated glass substrates were cleaned and treated with O_2_ plasma at 100 W for 60 s. The ZnO precursor solution was spin-coated onto the substrate at 3000 rpm for 40 s and then annealed at 200 °C for 1 h, forming a ZnO layer with a thickness of 30 nm [22].

For the active layer, hybrid solutions were prepared using PTB7-Th (1-material, Dorval, QC, Canada), PC_71_BM (1-material, Dorval, QC, Canada), and InP/ZnSe/ZnS QDs at a weight ratio of 1:1.5:(0, 0.2, 0.4, 0.6, 0.8, 1.0) (*w*/*w*/*w*) (Table 1). PTB7-Th and PC_71_BM were used as received without any further purification. The total solid content was 25 mg/mL, dissolved in 1 mL of chlorobenzene (Sigma-Aldrich, St. Louis, MO, USA) with 30 µL of diiodoctane (DIO) (Sigma-Aldrich, St. Louis, MO, USA) as an additive [23,24]. The solution was stirred overnight at room temperature and filtered through a 0.45 µm PVDF filter. Spin-coating was performed inside a nitrogen-filled glovebox at 1000 rpm for 90 s, followed by drying at room temperature for 2 h. Spin-coating at 1000 rpm was selected based on prior literature [24,25,26,27,28,29], which shows that this condition reliably yields films of approximately 100 nm thickness with stable optoelectronic performance, particularly in terms of minimizing dark current while maintaining EQE. A series of hybrid batches (H1–H6) were prepared by fixing the PTB7-Th donor content at 1 wt-equiv and the PC_71_BM acceptor at 1.5 wt-equiv, while systematically increasing the loading of InP/ZnSe/ZnS QDs from 0 to 1.0 wt-equiv. H1 was used as the QD-free control, whereas H2–H6 enabled a systematic evaluation of the effect of QD content on the device performance. A 10 nm layer of MoO_3_ followed by a 120 nm Ag electrode was sequentially deposited via thermal evaporation under high vacuum to form the top electrode.

## 3. Results and Discussion

### 3.1. Characterization

Previous studies [23,30,31,32,33,34] have shown that excessive PC_71_BM loading (>1:2) leads to disrupted crystallinity, reduced domain size, and vertical phase separation, which degrade carrier extraction and overall performance. Based on these findings, we adopted a 1:1.5 donor–acceptor ratio, which has been reported to yield optimal morphology and charge transport in PTB7-Th:PC_71_BM systems.

### 3.2. Photophysical Properties of InP/ZnSe/ZnS QDs in BHJ Matrix

**Figure 3:**
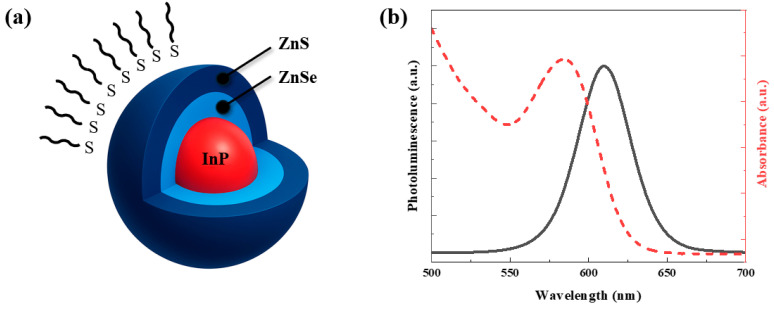
(**a**) Three-dimensional schematic of a dodecanethiol-capped InP/ZnSe/ZnS core–shell—shell quantum dot. (**b**) Normalised optical fingerprints of the InP/ZnSe/ZnS quantum dots: photoluminescence (black solid) and UV–Vis absorbance (red dashed).

Figure 3a schematically illustrates a red InP core with a ZnSe intermediate shell and a ZnS outer shell with dodecanethiol (DDT) ligands uniformly oriented on the outer surface via sulfur (S) atoms. This core–shell-shell configuration passivates the surface defects in the InP core, thereby enhancing the luminescence quantum efficiency [17]. The long alkyl chains of DDT improved the compatibility with the hydrophobic matrix and further suppressed the interfacial states, effectively reducing charge recombination. Thus, the DDT-capped InP/ZnSe/ZnS QDs serve as crucial building blocks for achieving a low dark current and improved photoelectric conversion efficiency in BHJ systems. The synthesis route and photoluminescence quantum yield (PLQY) of the InP/ZnSe/ZnS QDs are provided in Supplementary Section S1.

To quantitatively characterize the intrinsic optical properties of the QDs, the normalized absorption (red dashed line) and photoluminescence (black solid line) spectra of the InP/ZnSe/ZnS nanocrystals were measured and compared, as shown in Figure 3b. The absorption spectrum exhibits a broad feature with a primary peak near 525 nm [35], corresponding to the 1s-1s transition, and a shoulder around 560 nm, indicating a uniform size distribution and well-defined electronic states in the core–shell structure. In contrast, the PL spectrum exhibits a single emission peak at approximately 610 nm with a full width at half maximum (FWHM) of approximately 30 nm, reflecting a narrow size dispersion and high internal quantum efficiency. The Stokes shift of approximately 50 nm between the absorption and emission peaks minimizes reabsorption losses and spectrally aligns with the 600–650 nm region, which is critical for efficient photoconversion. These optical characteristics are expected to improve the photoelectric performance of the hybrid BHJ devices incorporating QDs.

### 3.3. UV–Vis Absorption and Photoluminescence Quenching in PTB7-Th:InP/ZnSe/ZnS QDs Hybrid Films

**Figure 4:**
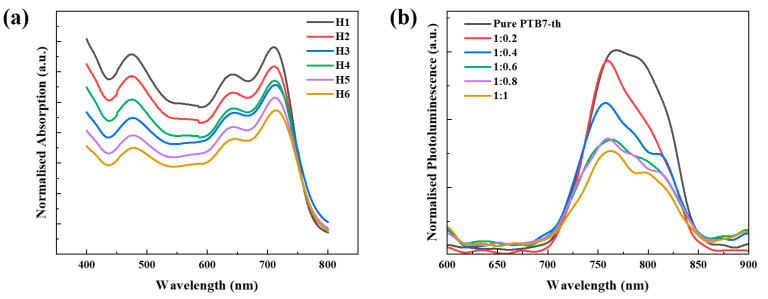
Optical characterization of PTB7-Th:PC_71_BM:InP/ZnSe/ZnS QDs hybrids: (**a**) normalised UV–Vis absorbance spectra of full BHJ films for increasing QD loadings (H1–H6); (**b**) normalised photoluminescence spectra of PTB7-Th  +  QD blends (polymer:QD = 1:x) showing progressive quenching with higher QD fractions.

UV-Vis absorption and PL spectroscopy were conducted to quantitatively assess the optical changes in the hybrid thin films. As shown in Figure 4a, the normalized absorption spectra revealed a gradual attenuation in intensity across the visible range as the QD content increased from H1 to H6. This decrease is attributed to a reduced integrated absorption coefficient stemming from the lower effective optical thickness of the donor–acceptor network upon QD incorporation, quantitatively indicating a loss in the light-harvesting capacity. Notably, the position of the characteristic peak near 700 nm and the overall spectral shape remained largely unchanged, suggesting that the polymer–fullerene molecular interactions were preserved despite hybridization.

Figure 4b shows the normalized PL spectra of the PTB7-Th thin films containing increasing amounts of InP/ZnSe/ZnS QDs with mass ratios ranging from 1:0.2 to 1:1. As the QD concentration increased, the PL intensity near 750 nm progressively decreased, with a reduction of more than 70% at the highest QD loading (1:1) compared with pristine PTB7-Th. This pronounced quenching is attributed to suppressed radiative recombination at the QD–polymer interface [9], likely due to enhanced exciton dissociation or transfer to nonradiative pathways. These findings suggest that QD incorporation facilitates charge separation by reducing luminescence losses, thereby improving device performance in hybrid systems.

### 3.4. Electrical Characteristics of PTB7-Th:PC_71_BM:InP/ZnSe/ZnS QDs Hybrid OPDs

**Figure 5:**
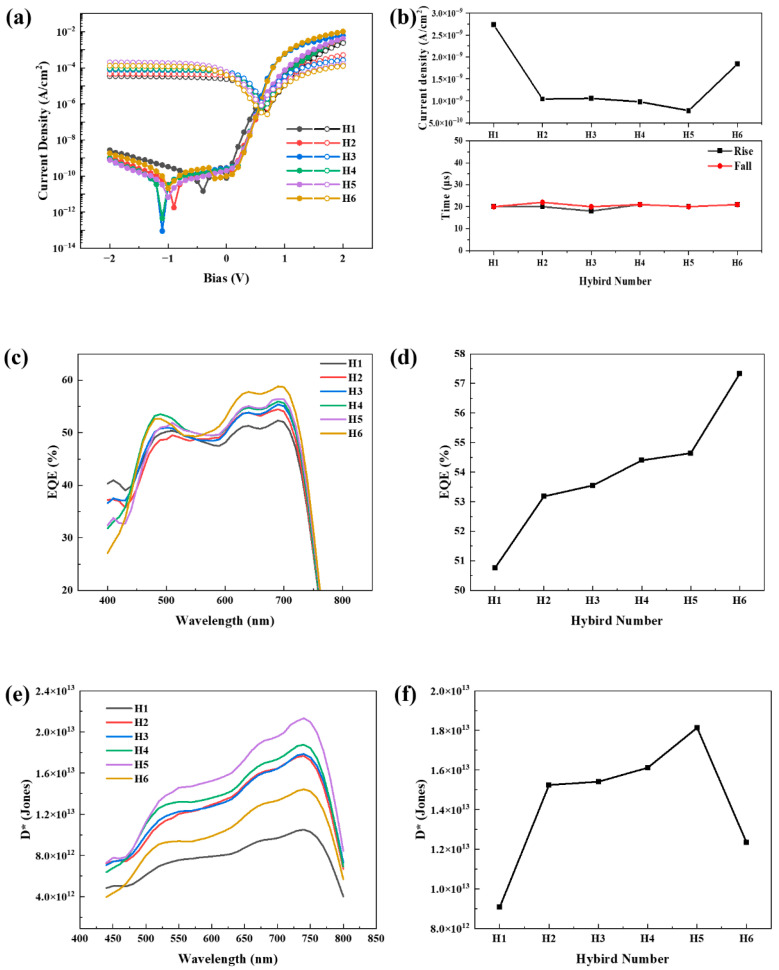
Electrical and optoelectronic performance of PTB7-Th:PC_71_BM:InP/ZnSe/ZnS QDs hybrid photodetectors as a function of QD loading. (**a**) Log scale J–V characteristics measured for H1–H6 over −2 V ≤ V ≤ +2 V: dark current densities are plotted with filled circles, while photocurrents under 660 nm illumination are shown with open circles, (**b**) −2 V dark-current density and 10–90% rise/fall time as a function of hybrid number, (**c**) EQE spectra measured at −2 V, (**d**) EQE at 660 nm, (**e**) specific D* spectra, and (**f**) D* at 660 nm plotted versus hybrid number.

The effect of increasing the InP/ZnSe/ZnS QDs content in the hybrid active layer is illustrated in Figure 5. As depicted in Figure 5a, the dark-current traces (filled circle) at a reverse bias of −2 V decrease significantly from H2 onward, reaching a minimum value of approximately 7.76 × 10^−10^ A/cm^2^ at H5. Under the same bias, the photocurrent traces (open circles) recorded under 660 nm illumination are not only preserved but rise to a peak of 1.995 × 10^−4^ A/cm^2^ at H5, confirming that QD incorporation enhances photo-generated charge collection while suppressing leakage. This reduction in dark current density (*J**_d_***) is attributed to the QDs effectively curbing the leakage current and promoting balanced charge transport. However, at a higher QD loading (H6), *J**_d_*** rises again, highlighting the existence of an optimal doping window. Figure 5b compares the dark current density extracted at −2 V and the 10–90% rising/fall time obtained from the transient response measurement for each hybrid composition. The rising/fall time remains almost constant at around 20 µs for all samples, indicating that the PTB7-Th:PC_71_BM matrix has sufficiently fast response speeds and that QD doping does not significantly improve speed. Figure 5c plots the EQE spectra of the hybrid devices under a −2 V bias. With increasing QD content, a marked enhancement in the EQE is observed within the 600–700 nm range, particularly at approximately 660 nm. Notably, this enhancement persists across the full 400–800 nm region, despite a reduction in the overall light absorption. As shown in Figure 5d, the EQE at 660 nm, a critical wavelength for PPG sensing, increases nearly linearly from 51% (H1) to 57% (H6), indicating that QD incorporation enhances the charge generation and extraction efficiency even as the optical density decreases.

The simultaneous enhancement of EQE and suppression of dark current directly translates to improved specific D* that was calculated as (*EQE*/1240 × *λ*)/(2*qJ**_d_***)^0.5^, where λ is the target wavelength and *q* is the electric charge [36]. As shown in Figure 5e, H5 exhibits the highest D* values across the 450–750 nm range, indicating an optimal tradeoff between charge generation and leakage suppression. At 660 nm (Figure 5f), a key wavelength for PPG sensing, D* reaches 1.84 × 10^13^ Jones for H5, more than double that of the QD-free reference H1 (9.56 × 10^12^ Jones). In contrast, D* decreased in H6 owing to a resurgence in the dark current at excessive QD loading. These results demonstrate that dark current suppression, EQE enhancement, and high detectivity can be simultaneously achieved by optimizing QD concentration under a −2 V bias. The H5 composition represents the most favorable convergence point for designing high-sensitivity organic photodiodes suitable for PPG applications.

**Figure 6:**
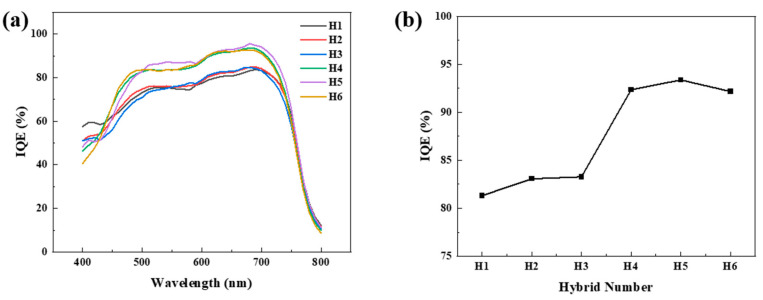
Internal quantum efficiency of PTB7-Th:PC_71_BM:InP/ZnSe/ZnS QDs hybrid photodetectors: (**a**) spectrally resolved IQE spectra (H1–H6, −2 V); (**b**) IQE extracted at 660 nm plotted as a function of hybrid number.

Figure 6 characterizes the effect of the InP/ZnSe/ZnS QD content in the hybrid active layer on the IQE. Figure 6a presents the spectral IQE measured under a −2 V reverse bias condition. The reference sample (H1), which did not contain QDs, exhibited a response level of 60–80% across the 400–800 nm region. However, as the QD content increased, the IQE increased across the spectrum. This improvement was particularly evident in the 600–700 nm region, where the wavelength dependence flattened for H4 and H5, approaching a maximum value of approximately 95%. This outcome is interpreted as the QDs streamlining the charge generation, separation, and collection processes, significantly reducing the photoelectric conversion losses despite the reduced total absorption coefficient. Conversely, a slightly lower IQE was observed for H6, which had an excessively high QD ratio, suggesting that the efficiency decreased when the optimal concentration range was exceeded [37]. Figure 6b shows the IQE extracted at the 660 nm wavelength used for PPG sensing plotted against the hybrid number. The value of 81% at H1 increased linearly with the QD content, reaching 93–94% at H4 and H5 and decreasing slightly to 92% at H6. This trend demonstrates that the optimal QD concentration maximizes the charge-collection efficiency, substantially improving the photoconversion performance at specific wavelengths. Therefore, the H4–H5 composition provides the most favorable IQE–photo response tradeoff for designing highly sensitive OPDs for PPGs.

A slight reduction in total light absorption upon QD doping was observed (Figure 4a). Nevertheless, the marked enhancement in EQE (Figure 5c) can be attributed to the improved IQE, which increased from 80% to approximately 95% at 0.8 wt% QD doping (Figure 6). Given that EQE is the product of absorption and IQE (EQE = Absorption × IQE), the gain in IQE effectively compensates for the reduced absorption. These findings indicate that QDs facilitate efficient charge separation and transport, thereby minimizing photoconversion losses and playing a critical role in determining the electrical performance of the device.

### 3.5. Surface Morphology of the PTB7-Th:PC_71_BM:InP/ZnSe/ZnS QD BHJ Film

**Figure 7:**
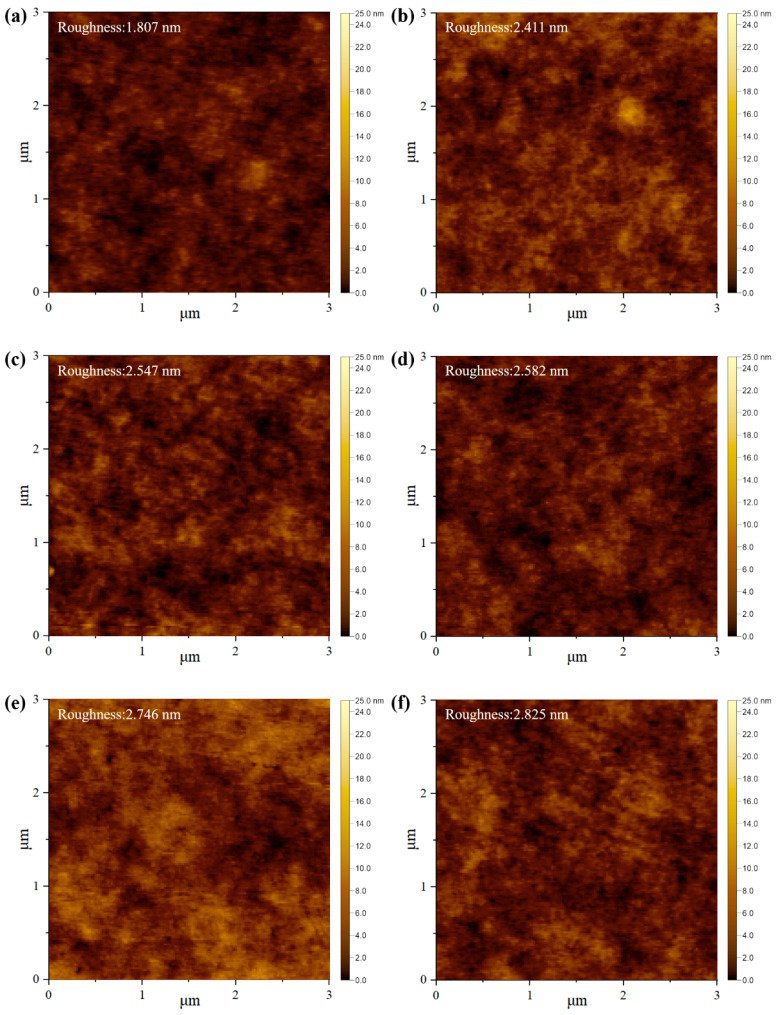
3 × 3 µm^2^ AFM height images of PTB7-Th:PC_71_BM:InP/ZnSe/ZnS QD hybrids with increasing QD loading: (**a**) H1, (**b**) H2, (**c**) H3, (**d**) H4, (**e**) H5 and (**f**) H6. RMS roughness steadily rises from 1.807 nm to 2.825 nm.

To further elucidate the structure–property relationships within the active layer, atomic force microscopy (AFM) height imaging was conducted on the PTB7-Th:PC_71_BM:QD films with increasing QD content. Figure 7a–f present the corresponding AFM height images acquired over a 3 μm × 3 μm scan area. The root mean square (RMS) roughness values gradually increased with QD loading, measured as 1.807 nm (H1), 2.411 nm (H2), 2.547 nm (H3), 2.582 nm (H4), 2.746 nm (H5), and 2.825 nm (H6). Despite the gradual increase in surface roughness, the AFM images show no evidence of large grain boundaries or isolated insulating domains, suggesting that the addition of QDs enhances nanoscale phase separation between polymer and fullerene components without compromising the interpenetrating network of the BHJ. This controlled morphological evolution is beneficial for exciton dissociation and charge transport, as it maintains continuous pathways for carrier extraction while promoting effective spatial organization of the donor and acceptor phases. As shown in Figure 4b, the enhanced PL quenching observed with QD incorporation suggests that excitons generated in the p-type semiconductor are effectively transferred to the QDs, facilitating efficient charge separation. This implies that QD doping does not deteriorate charge separation efficiency but rather contributes to improved charge transport. While not providing direct experimental validation, the combination of increased IQE, morphological analysis, and PL quenching behavior supports the conclusion that the introduction of QDs plays a role in suppressing interface traps, thereby reducing internal losses and enhancing overall device performance.

### 3.6. Photoplethysmographic Signal Acquisition and SNR Enhancement

**Figure 8:**
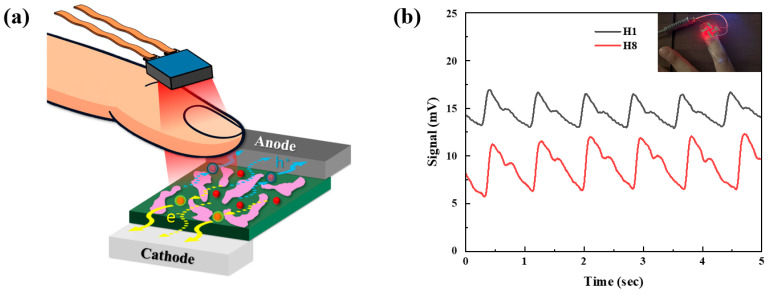
(**a**) Schematic illustration of the pulse oximetric sensing system in a transmissive configuration. (**b**) Five-second PPG time-domain traces measured with a TI AFE4490 module under 660 nm illumination: reference H1 (black) versus QD-optimized H5 (red).

As illustrated in Figure 8a, pulse oximetric sensing was performed in a transmissive configuration, where a 660 nm LED illuminated the fingertip and the hybrid photodiode, placed on the opposite side, detected the modulated light signal. The schematic also depicts exciton dissociation and charge transport pathways (e^−^/h^+^) facilitated by the uniformly dispersed InP/ZnSe/ZnS QDs, emphasizing their contribution to efficient carrier extraction. We compared the raw voltage signals acquired from the hybrid photodiode samples, reference H1, and QD-optimized H5, under 660 nm LED illumination using Texas Instruments’ AFE4490 PPG module. The sampling rate was set at 500 Hz. Figure 8b plots the time-domain waveform over a 5-s interval, showing that the H5 device exhibits a higher average peak amplitude and improved slope resolution compared to the H1 device. Subsequently, the SNR was calculated for the frequency domain. First, the power spectrum was obtained via a fast Fourier transform (FFT). The spectral power *P_sig_* in the pulse-wave frequency band (0.5–10 Hz) and the noise power *P_noise_* in the frequency range above 10 Hz were extracted. The SNR was calculated using the following equation [38]:

(1) $$SNR(dB)=10\log_{10}\left(\frac{P_{sig}}{P_{noise}}\right)$$

The calculated SNRs for H1 and H5 were 15 and 19 dB, respectively. This approximate enhancement of 4 dB (corresponding to a power gain of approximately 60%) confirms that the previously reported suppression of dark current and enhancement in EQE and D* at 660 nm effectively translate into practical PPG measurement environments.

## 4. Conclusions

In this work, we presented an organic photodetector optimized for 660 nm-based PPG sensing by precisely doping Cd-free InP/ZnSe/ZnS QDs into a PTB7-Th:PC_71_BM BHJ. The hybrid device (H5), containing a moderate QD concentration, exhibited enhanced external and internal quantum efficiencies of 57% and 94%, respectively, at 660 nm, while suppressing the dark current density from 2.7 × 10^−9^ to 7.76 × 10^−10^ A/cm^2^. These improvements in photovoltaic performance approximately doubled the specific D*, increasing it from 9.56 × 10^12^ to 1.84 × 10^13^ Jones. In practical PPG measurements, the SNR improved from 15 to 19 dB, leading to a notable enhancement in the pulse waveform clarity. The appropriate incorporation of QDs effectively reduced the leakage current with minimal sacrifice of the photoelectric conversion efficiency, demonstrating the potential of high-sensitivity, low-power, and wearable Cd-free hybrid OPDs for pulse-wave sensing. Among the tested devices, the mid-level QD content (H5) was found to be optimal for simultaneously maximizing the dark current suppression and promoting efficient photogenerated charge separation, thereby enhancing both D* and PPG-SNR. This study demonstrated the practical applicability of flexible low-power OPDs that incorporate Cd-free QDs in wearable PPG sensor systems. Further improvements in device sensitivity and reliability are expected through fine control of the QD surface chemistry and orientation.

## Figures and Tables

**Table 1:** Composition of PTB7-Th:PC_71_BM:InP/ZnSe/ZnS QDs Hybrid Blends (weight/weight/weight).

Hybird Number	PTB7-Th (*w*/*w*/*w*)	PC_71_BM (*w*/*w*/*w*)	InP/ZnSe/ZnS QDs (*w*/*w*/*w*)
H1	1	1.5	0
H2	1	1.5	0.2
H3	1	1.5	0.4
H4	1	1.5	0.6
H5	1	1.5	0.8
H6	1	1.5	1.0

## Data Availability

The original contributions of this study are included in this article. Further inquiries can be directed to the corresponding authors.

## Supplementary Materials

The following supporting information can be downloaded at: https://www.mdpi.com/article/10.3390/polym17162214/s1, Figure S1: PLQY spectrum of InP/ZnSe/ZnS QDs.
