# Supplementary material for: High-Detectivity Organic Photodetector with InP Quantum Dots in PTB7-Th:PC71BM Ternary Bulk Heterojunction
Source: Polymers (Basel). 2025 Aug 13;17(16):2214. doi: 10.3390/polym17162214 (PMC12389295; doi:10.3390/polym17162214)
Supplement: Supplementary file 1 [file polymers-17-02214-s001.zip › polymers-3780177-supplementary.pdf]

S1. Synthesis and Characterization of InP/ZnSe/ZnS Core–Shell–Shell Quantum Dots

InP core quantum dots (QDs) with an emission peak at 570 nm were synthesized and subsequently coated with ZnSe and ZnS shells via a two-step hot-injection method under a nitrogen atmosphere using a standard Schlenk line setup. For the ZnSe shell formation, a $Zn(OA)_2$ precursor—prepared from zinc acetate, oleic acid, and trioctylamine (TOA)—was sequentially injected along with a small amount of TOP-Se into the TOA solution containing InP cores. After the initial injection, the mixture underwent hydrofluoric acid (HF, 0.1 mL) treatment to remove native surface oxides. The reaction temperature was then raised to 220 °C and maintained for 30 minutes to form a thin Se interfacial layer, followed by an additional TOP-Se injection to complete the ZnSe shell growth. ZnS shelling was carried out in two steps: the first injection of TOP-S (1.6 mmol) at 320°C and a second injection (3.39 mmol) at 280°C. The resulting core–shell–shell QDs were purified by centrifugation and redispersed in toluene at a concentration of 100 mg/mL for further use.

The synthesized InP/ZnSe/ZnS core–shell–shell QDs exhibit narrow-band photoluminescence centered at 608 nm with a high photoluminescence quantum yield (PLQY) of 95.7 ± 1.4% and a full width at half maximum (FWHM) of 39.0 ± 0.1 nm.

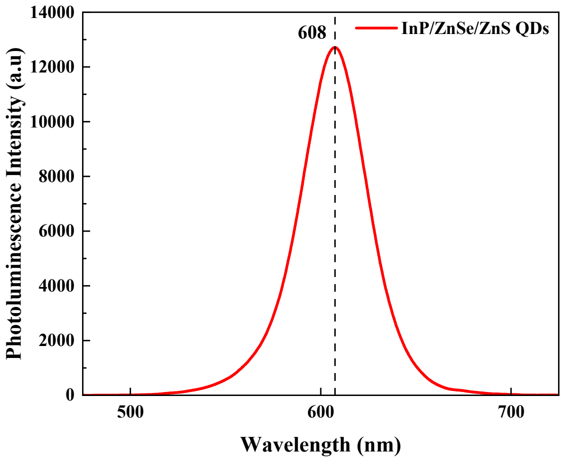

Figure S1. PLQY spectrum of InP/ZnSe/ZnS QDs
